# Alopecia in Patients with Collagen VI-Related Myopathies: A Novel/Unrecognized Scalp Phenotype

**DOI:** 10.3390/ijms24076678

**Published:** 2023-04-03

**Authors:** Michela Starace, Francesca Pampaloni, Francesca Bruni, Federico Quadrelli, Stephano Cedirian, Carlotta Baraldi, Cosimo Misciali, Alberto Di Martino, Patrizia Sabatelli, Luciano Merlini, Bianca Maria Piraccini

**Affiliations:** 1 Dermatology Unit, IRCCS Azienda Ospedaliero-Universitaria di Bologna, 40138 Bologna, Italy; 2 Department of Medical and Surgical Sciences, Alma Mater Studiorum University of Bologna, 40126 Bologna, Italy; 3 Department of Biomedical and Neuromotor Sciences, University of Bologna, 40126 Bologna, Italy; 4 Clinica Ortopedica e Traumatologica I, IRCCS Istituto Ortopedico Rizzoli, 40136 Bologna, Italy; 5 Sidney Kimmel Medical College, Thomas Jefferson University, Philadelphia, PA 19107, USA; 6 Unit of Bologna, CNR-Institute of Molecular Genetics “Luigi Luca Cavalli-Sforza”, 40136 Bologna, Italy; 7 IRCCS Istituto Ortopedico Rizzoli, 40136 Bologna, Italy

**Keywords:** collagen type VI, COL VI-related myopathies, alopecia, scalp disorder

## Abstract

Collagen VI-related myopathies are characterized by severe muscle involvement and skin involvement (keratosis pilaris and impaired healing with the development of abnormal scars, especially keloids). Scalp involvement and hair loss have not been reported among cutaneous changes associated with collagen VI mutations. The aim of this study is to describe the clinical, trichoscopic, and histological findings of the scalp changes in patients affected by COL VI mutations and to estimate their prevalence. Patients with Ullrich congenital muscular dystrophy were enrolled and underwent clinical and trichoscopic examinations and a scalp biopsy for histopathology. Five patients were enrolled, and all complained of hair loss and scalp itching. One patient showed yellow interfollicular scales with erythema and dilated, branched vessels, and the histological findings were suggestive of scalp psoriasis. Two patients presented with scarring alopecia patches on the vertex area, and they were histologically diagnosed with folliculitis decalvans. The last two patients presented with scaling and hair thinning, but they were both diagnosed with folliculitis and perifolliculitis. Ten more patients answered to a “scalp involvement questionnaire”, and six of them confirmed to have or have had scalp disorders and/or itching. Scalp involvement can be associated with COL VI mutations and should be investigated.

## 1. Introduction

Collagen VI (COL VI) is a fundamental component of the extracellular matrix (ECM) and has been studied in the past few years in a wide range of tissues, including muscles, tendons, cartilage, and skin. In humans, it is encoded by five different genes: *COL6A1*, *COL6A2*, *COL6A3*, *COL6A5*, and *COL6A6* [1,2]. Collagen VI is expressed as an ubiquitous ECM protein in the stroma of tissues. Ultrastructurally, collagen VI forms a network of beaded microfilaments anchored to the cell surface [2,3] that connects the cell cytoskeleton to the extracellular environment by direct interaction with several ECM constituents, including fibrillar collagens I and II, collagen IV, fibronectin, decorin, and biglycan [4,5,6]. The best-characterized and most widely expressed form of collagen VI is the (α1, α2, and α3) heterotrimer. The α5 and α6 chains are homologues of the α3 chain but display a more restricted and differential distribution pattern. The ubiquitous expression of collagen VI, and its interaction with the ECM constituents, suggest that it is involved in the development of the ECM supramolecular arrangement, playing a key role in repairing processes and tissue development [2,6]. Mutations in the *COL6A1*, *COL6A2*, and *COL6A3* genes cause collagen VI-related myopathies (COL6-RM) that include Ullrich congenital muscular dystrophy (UCMD), Bethlem myopathy (BM), and myosclerosis myopathy (MM) [7].

UCMD is the most severe and progressive form of collagen VI-related myopathies and results from either recessive or dominant mutations. It presents at birth with hypotonia, muscle weakness, and a combination of proximal contractures and distal laxity. Patients affected by UCMD do not acquire independent ambulation or lose it in the first 12 years of life and develop a severe respiratory involvement requiring ventilatory support in the first or second decade of life [7]. Bethlem myopathy is a milder disorder, with variable onset from congenital to adult age, characterized by axial and proximal muscle weakness; variable contractures of the neck, fingers, elbows, knees, and ankles; distal laxity; and skin changes. Progression is also variable, and some individuals require supportive means for outdoor mobility [8]. There are also patients with a clinical severity in between UCMD and Bethlem, who lose ambulation during their teens and are reported as an intermediate phenotype [7]. Myosclerosis myopathy is characterized by early and progressive contractures of all joints, including limitation of mouth opening [9]. Patients affected by collagen VI mutations can also present skin involvement. The most-reported skin manifestations are keratosis pilaris on the extensor surfaces of proximal legs and arms; soft and velvet skin on palms and soles; impaired healing with the development of abnormal scars, in particular keloids, that can appear spontaneously or either after minor trauma; rough or dry skin; stria rubrae; and cigarette paper scars [10,11,12,13]. Hair loss is not reported among skin changes associated with collagen VI mutations. However, five patients of the Italian Collagen VI group (https://www.col6.it/) contacted us complaining of hair loss and an itchy scalp, which was a source of severe discomfort. The complaints of these five patients led us to send a brief “scalp involvement questionnaire” to the adult patients with COL6-RM attending the Rizzoli Orthopedic Institute and the Department of Biomedical and Neuromotor Sciences of the University of Bologna to find out if other patients reported hair problems. Surprisingly, we found out that the scalp involvement is common in the COL6-RM population and decided to start to evaluate these problems in the first five patients. 

The aim of this study is to report the clinical, trichoscopic, and skin biopsy findings of the scalp changes in patients affected by COL6-related myopathies and to estimate their prevalence. 

## 2. Results

### 2.1. Demographic and Clinical Characteristics

Five patients affected by Ullrich congenital muscular dystrophy were included in the study. The demographic, genetic, clinical, and histological characteristics of patients are summarized in Table 1. The mean age was 27.8 (±5.54), four were females, and one was male. All subjects showed keloids and follicular hyperkeratosis, and they also complained about hair loss and severe scalp itching; only one patient defined the itching as “mild”.

### 2.2. Trichoscopic and Histological Findings

**Figure 1:**
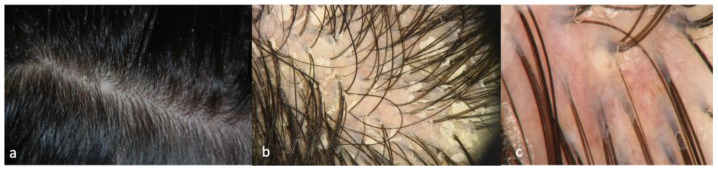
Clinical (**a**) and trichoscopic findings of Patient 1, (**b**) 20× magnification, and (**c**) magnification 40×.

**Figure 2:**
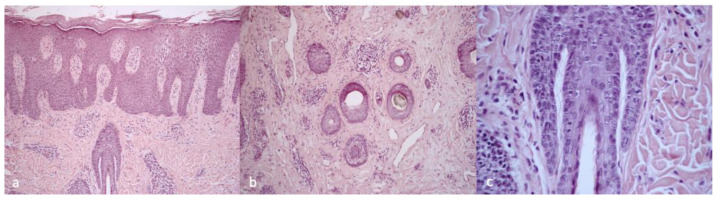
Histological findings of Patient 1. (**a**) Parakeratosis, focal absence of granular layer, neutrophilic infiltrate in the papillary dermis and mantles (sebaceous structures), H&E 10× magnification; (**b**) dilated vessels in the dermis and neutrophilic infiltrate, H&E 10× magnification; and (**c**) high magnification of mantles (sebaceous structures), H&E 40× magnification.

Patient 1 clinically presented scalp itching and desquamation that at trichoscopy appeared as yellow interfollicular scales with erythema and dilated, branched vessels (Figure 1). The histological findings were suggestive of scalp psoriasis with epidermal parakeratosis and a focal absence of granular layer with a neutrophilic infiltrate and dilated vessels in the dermis and mantles (sebaceous structures) (Figure 2).

**Figure 3:**
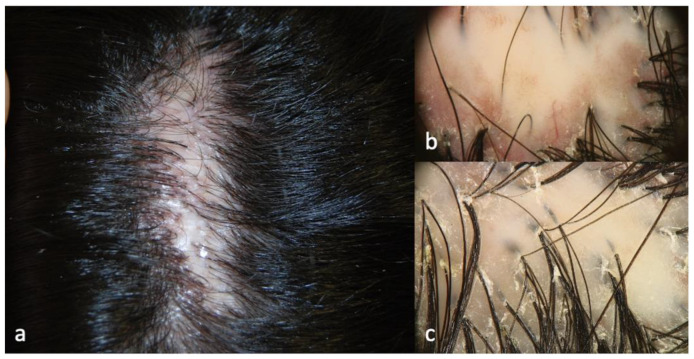
Clinical (**a**) and trichoscopic findings of Patient 2 and (**b**,**c**) magnification 20×.

**Figure 4:**
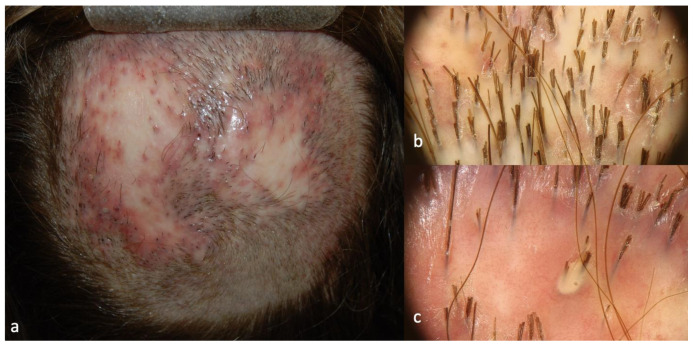
Clinical (**a**) and trichoscopic findings of Patient 3, (**b**) magnification 20×, and (**c**) magnification 40×.

**Figure 5:**
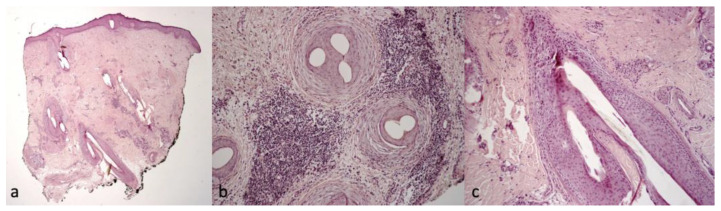
Histological findings of Patients 2 and 3, showing diffuse fibrosis, focal perifollicular suppurative, and mixed infiltrate in the dermis. “Tufted folliculitis” (**a**) 2× magnification, H&E and (**b**,**c**) 20× magnification, H&E.

Patients 2 and 3 showed similar scalp signs; the first presented with a linear band of alopecia in the vertex area, 5 × 2 cm in size, with white skin completely devoid of hair in the center and erythema at the periphery (Figure 3a); the second patient showed a wider cicatricial area made by two confluent patches, sized around 7 × 5 cm and 3 × 4 cm on the vertex. The scarring area was surrounded by severe erythema and pustules localized along the edge of the patch (Figure 4a). The trichoscopy of Patient 2 (Figure 3b,c) showed an absence of follicular ostia in the center, a suggestive sign of cicatricial alopecia, and the presence of hair tufting (groups of 5–6 hairs emerging from the same follicular ostium) at the periphery with scaling and hair casts (cylindrical formation of scales surrounding the hair shaft); the trichoscopic findings in Patient 3 (Figure 4b,c) included the absence of follicular ostia in the scarring area, severe erythema, dilated vessels, yellow scales, tufted hair, and perifollicular pustules. The histopathological analysis of Patients 2 and 3 was consistent with the diagnosis of folliculitis decalvans, showing loss of hair follicles with a diffuse dense suppurative, mixed infiltrate in the dermis, associated with follicular tufting (two or three follicles sharing the same infundibulum) and intraepidermal pustules (Figure 5).

Patient 4 complained about severe itching, and the clinical and trichoscopic evaluations showed fine perifollicular scaling. The clinical presentation of Patient 5 showed slight hair thinning, confirmed also by trichoscopic evaluation, which highlighted the presence of hair diameter variability (anysotrichosis), erythema, and yellow scales.

**Figure 6:**
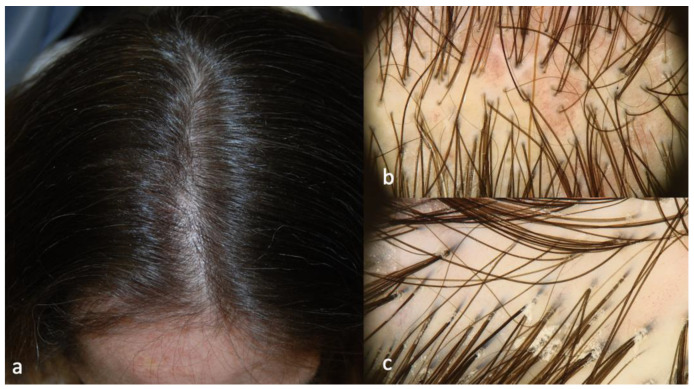
Clinical (**a**) and trichoscopic findings of Patient 4 and (**b**,**c**) magnification 20×.

**Figure 7:**
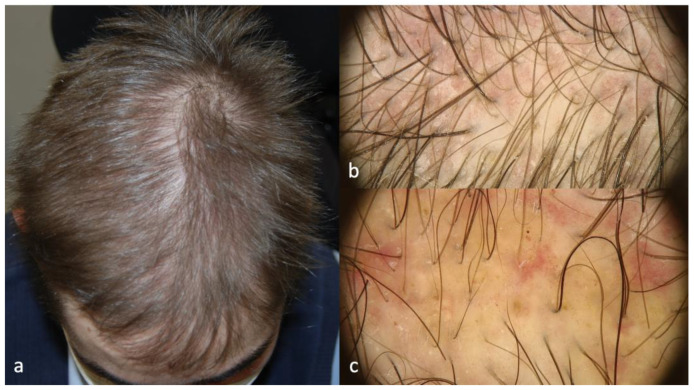
Clinical (**a**) and trichoscopic findings of Patient 5 and (**b**,**c**) magnification 20×.

**Figure 8:**
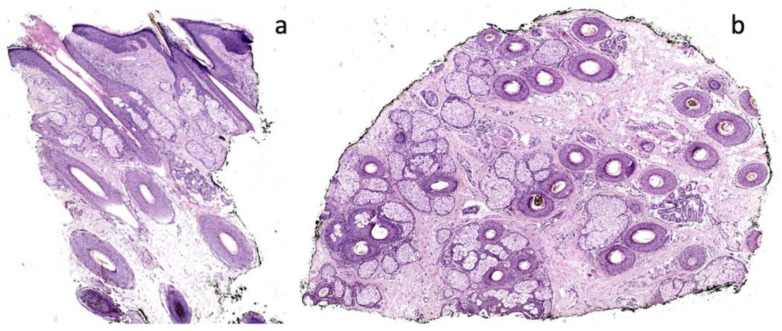
Histological findings typical of folliculitis and perifulliculitis (Patients 4 and 5). (**a**) 2× magnification, H&E and (**b**) 10× magnification, H&E.

Patients 4 and 5 showed variable clinical and trichoscopic findings (Figure 6b,c and Figure 7b,c), but the same histopathological analysis, which was consistent with folliculitis and perifolliculitis, showed superficial perivascular and perifollicular infiltrates of lymphocytes and some neutrophils (Figure 8).

No involvement of body hair was detected in any patient, neither on the eyebrows nor eyelashes.

Ten patients, beyond those five enrolled, responded to the questionnaire that was sent by e-mail, and six out of them confirmed to have or have had scalp disorders and/or itching problems and were willing to be contacted by a dermatologist. Of these six patients, one showed an intermediate phenotype, with a *COL6A2*, c.875G>C, and p.Gly292Ala genomic mutation; while five were affected by Bethlem myopathy with the following genomic mutations: *COL6A2*, c.883G>A, and p.Gly295Arg; *COL6A3*, c.6158G>T, and p.Gly2053Val; *COL6A1*, c.428+1G>A, and Tyr122-Gly143del; *COL6A2*, c.847G>A, and p.Gly283Arg; and *COL6A2*, c.802G>A, and p.Gly268Ser.

## 3. Discussion

Collagen VI-related myopathies are characterized by hypotonia, muscles weakness, proximal contractures, and distal laxity as collagen VI is an ECM protein critical in maintaining the integrity and function of muscles and skin [7]. Several skin disorders, such as abnormal scars, follicular hyperkeratosis, keloids, rough skin, and cigarette scars, have been described in patients affected by the genetic mutation of collagen VI [10,11,12,13], but scalp problems and alopecia have never been reported. We described the clinical presentations, trichoscopic characteristics, and histological findings of scalp involvement in five patients affected by a defect of collagen VI. All patients started to complain about severe itching of the scalp and different forms of localized alopecia, and they all underwent trichoscopy and a skin biopsy. One of the five subjects, (Patient 1) was diagnosed with psoriasis, which is a chronic inflammatory disorder that may involve the scalp. Scalp psoriasis is seen in about 80% of patients affected by psoriasis, but in about 25% of patients, the scalp can remain the unique location [14]. Scalp psoriasis is characterized by itching and scaling and by the following trichoscopic findings: yellow interfollicular scales, erythema, and twisted red loop vessels as in the reported patient [14]. The patient did not show any other skin signs of psoriasis, and no other family member was affected. Moreover, collagen VI and, in particular, the COL6A5 protein, is responsible for focal adhesion and phosphatidylinositol-3-kinase (PI3K)/AKT Serine/Threonine Kinase (AKT) pathways, thus influencing cell survival and proliferation. This suggests a potential contribution of COL6A5 to the etiopathogenesis of psoriasis [15]. Patients 2 and 3 were diagnosed with folliculitis decalvans. Folliculitis decalvans (FD) of Quinquaud is a rare form of neutrophilic scarring alopecia, first described in 1888 as a chronic inflammatory disease that usually affects young adults of both genders. The pathogenesis is still poorly defined, but the Staphylococcus aureus infection and a dysfunction of the patient’s immune response seem to play an important role [16]. Folliculitis decalvans is clinically characterized by cicatricial alopecia surrounded by tufted folliculitis and at trichoscopy, by the absence of follicular ostia, hair tufting, white and milky-red areas, and elongated twisted blood capillary loops [17]. Lastly, two of the five patients were diagnosed with folliculitis and perifolliculitis. The clinical presentation of the two patients was different, varying from mild erythema to diffuse interfollicular scaling. Thus, the scalp was involved in 5 out of the 5 patients with COL VI mutations that we could study directly and in 6 out of the 10 patients who answered the questionnaire. All five cases studied are Ullrich congenital muscular dystrophy patients, but of the six patients that answered the questionnaire by e-mail, five were affected by Bethlem myopathy, and one showed an intermediate phenotype. Therefore, it is not yet possible to define whether this scalp phenotype is typical only for Ullrich patients and not for all patients with different COL VI mutations.

Itching was a common complain, with scalp inflammatory changes that in most of the cases led to follicular destruction by a neutrophilic infiltrate. To our knowledge, no other study has reported the clinical, trichoscopic, and histological findings of the scalp in patients affected by COL VI mutations yet. Several hypotheses have been made regarding the pathogenesis underlying other skin conditions related to COL VI mutations. A detailed description of the different isoforms in the skin of normal and COL VI-related myopathies has been previously reported [18,19], showing that collagens VI α5 and α6 are expressed in the dermis and most likely assemble with collagens VI α1 and α2. It supports the hypothesis of a possible contribution of collagens VI α5 and α6 to the development of the skin phenotypes. It has also been shown that the ultrastructure of dermal collagens in patients with UCMD is altered because of collagen VI mutations [10]. The gene expression of types I and VI collagen is activated in keloids showing excessive levels of collagens VI and I and an increase in the collagen I/collagen III ratio in keloids, which is triggered by the elevated expression of transforming growth factor-β [20].

To the best of our knowledge, this is the first study describing the clinical, trichoscopic, and histological characteristics of the scalp in patients affected by COL VI mutation. The main limitation of this pilot study is the small number of patients enrolled, but this is a rare neurological disease, and not all the patients reported scalp involvement. Moreover, another limitation is that we did not enroll pediatric patients because this pilot study was focused only on patients that complained about hair loss and scalp itching. A detailed study of collagen VI isoforms in the scalp has so far not been comprehensively undertaken, and although the number of cases is limited, considering the rarity of the disease, the findings suggest that this might be a possibly overlooked finding in collagen VI myopathies.

Further studies involving more COL IV patients comparing subjects without COL VI mutations and focusing on the underlying pathological mechanisms are needed to better address these signs and symptoms.

## 4. Materials and Methods

In this pilot study, we enrolled five patients with Ullrich congenital muscular dystrophy already characterized both clinically and genetically. Patients included were subjects over 18 years and male and female; all subjects signed an informed consent before undergoing the noninvasive and invasive procedures. The study was conducted in accordance with the principles of the Declaration of Helsinki, and the protocol was approved by the Ethics Committee of the Rizzoli Orthopedic Institute (CE 0007151). The evaluations performed included clinical and trichoscopic examinations of the scalp hair, eyebrows, and eyelashes and a scalp biopsy (using a 5 mm punch) for histopathological examination with hematoxylin–eosin (H&E) staining. All patients underwent a complete dermatologic assessment, focusing on already described cutaneous signs in collagen VI-mutated subjects and on all body hair. Trichoscopy is a noninvasive technique useful for the diagnosis and follow-up of hair and scalp disorders, allowing a rapid examination, magnification, and image record of hair changes [21,22]. Trichoscopic patterns of hair disorders are different, and they are useful to distinguish scarring from nonscarring alopecia. The choice of biopsy site was trichoscopy-guided, choosing the area where dermoscopy highlighted signs of disease. For an accurate histological study of scalp disease, a study of both vertical and horizontal sections was performed, allowing immediate detection of location and spread of the inflammatory infiltrate and an assessment of follicular density, anagen/telogen ratio, diameter and shape of the follicles, and percentages of vellus hairs and terminal follicles, which are easier to detect with horizontal sections. Transversal sections also allow observation of 100% of the follicles contained in the sample and thus make it possible to study diseases affecting only a small number of follicles. The specimens were observed in hematoxylin and eosin (H&E) staining.

## Figures and Tables

**Table 1:** Demographic, clinical, genetic, trichoscopic, and histological characteristics of patients.

	Age (year)/ Sex	Phenotype	Genomic Mutation	Other Cutaneous Signs	Scalp Symptoms	Scalp Clinical Findings	Trichoscopic Findings	Histological Findings
1	25/F	UCMD	*COL6A1* c.850G>A	Keloids, follicular hyperkeratosis	Severe itching	White-yellow scales	Erythema, twisted red loop vessels, and yellow interfollicular scales	Dilated vessels in the dermis; parakeratosis, focal absence of granular layer in the epidermis, and neutrophilic infiltrate in the papillary dermis mantles (sebaceous structures)
2	29/F	UCMD	*COL6A1* c.958-2A>G	Face and neck hirsutism, keloids	Severe itching	Small cicatricial areas on vertex	Tufted hair, absence of follicular ostia in cicatricial area, and dilated elongated vessels	Dense diffuse suppurative, mixed infiltrate in the dermis. “Tufted folliculitis” and intraepidermal pustules
3	24/F	UCMD	*COL6A2* homozygous c.348dup	Keloids	Mild itching	Cicatricial patch on vertex	Severe erythema, dilated vessels, yellow scales, tufted hair, perifollicular pustules, and cicatricial areas with absence of follicular ostia	Superficial perivascular and perifollicular infiltrates of lymphocytes and neutrophils
4	37/F	UCMD	*COL6A2* c.875G>T	Keloids, follicular hyperkeratosis	Severe itching	Fine scaling	Perifollicular scales and erythema	Superficial perivascular and perifollicular infiltrates of lymphocytes and neutrophils; collection of neutrophils within infundibula
5	24/M	UCMD	*COL6A1* c.930+189C>T	Keloids, follicular hyperkeratosis	Severe itching	Hair thinning	Different hair diameters, erythema, dilated vessels, and yellow scales	Superficial perivascular and perifollicular infiltrates of lymphocytes and neutrophils

F = female; M = male; y = years; UCMD = Ullrich congenital muscular dystrophy.

## Data Availability

Data are available on request due to privacy or ethical restrictions.
